# First report on the occurrence of *Vibrio cholerae* nonO1/nonO139 in natural and artificial lakes and ponds in Serbia: Evidence for a long‐distance transfer of strains and the presence of *Vibrio paracholerae*

**DOI:** 10.1111/1758-2229.13136

**Published:** 2023-02-13

**Authors:** Carmen Rehm, Kathrin Lippert, Alexander Indra, Stoimir Kolarević, Margareta Kračun‐Kolarević, Melanie Leopold, Sophia Steinbacher, Iris Schachner, Lena Campostrini, Alexandra Risslegger, Andreas H. Farnleitner, Claudia Kolm, Alexander K.T. Kirschner

**Affiliations:** ^1^ Division Water Quality and Health, Department of Physiology, Pharmacology and Microbiology Karl Landsteiner University of Health Sciences Krems Austria; ^2^ Institute for Hygiene and Applied Immunology – Water Microbiology Medical University Vienna Vienna Austria; ^3^ Interuniversity Cooperation Centre Water & Health Austria; ^4^ Institute für Medical Microbiology and Hygiene, Austrian Agency for Health and Food Safety Vienna Austria; ^5^ Institute for Biological Research ¨Siniša Stanković¨, National Institute of the Republic of Serbia, Department for Hydroecology and Water Protection University of Belgrade Belgrade Serbia; ^6^ Institute for Chemical, Environmental and Bioscience Engineering, Technische Universität Wien Vienna Austria

## Abstract

*Vibrio cholerae* are natural inhabitants of specific aquatic environments. Strains not belonging to serogroups O1 and O139 are usually unable to produce cholera toxin and cause cholera. However, non‐toxigenic *V. cholerae* (NTVC) are able to cause a variety of mild‐to‐severe human infections (via seafood consumption or recreational activities). The number of unreported cases is considered substantial, as NTVC infections are not notifiable and physicians are mostly unaware of this pathogen. In the northern hemisphere, NTVC infections have been reported to increase due to global warming. In Eastern Europe, climatic and geological conditions favour the existence of inland water‐bodies harbouring NTVC. We thus investigated the occurrence of NTVC in nine Serbian natural and artificial lakes and ponds, many of them used for fishing and bathing. With the exception of one highly saline lake, all investigated water‐bodies harboured NTVC, ranging from 5.4 × 10^1^ to 1.86 × 10^4^ CFU and 4.5 × 10^2^ to 5.6 × 10^6^ genomic units per 100 ml. The maximum values observed were in the range of bathing waters in other countries, where infections have been reported. Interestingly, 7 out of 39 fully sequenced presumptive *V. cholerae* isolates were assigned as *V. paracholerae*, a recently described sister species of *V. cholerae*. Some clones and sublineages of both *V. cholerae* and *V. paracholerae* were shared by different environments indicating an exchange of strains over long distances. Important pathogenicity factors such as *hlyA*, *toxR*, and *ompU* were present in both species. Seasonal monitoring of ponds/lakes used for recreation in Serbia is thus recommended to be prepared for potential occurrence of infections promoted by climate change‐induced rise in water temperatures.

## INTRODUCTION

*Vibrio cholerae* is a natural inhabitant of specific aquatic environments with low‐to‐moderate salinity. Aside from toxigenic *V. cholerae* serogroup O1 and O139 strains, which are the causative agents of cholera, non‐toxigenic *V. cholerae* strains (NTVC) belonging mostly to non‐O1/non‐O139 serogroups are emerging waterborne pathogens causing mild‐to‐lethal infections of susceptible persons via ingestion of contaminated sea food, drinking water consumption or during recreational activities (Vezzulli et al., 2020). Infections range from ear infections, wound infections, septicaemia to gastrointestinal infections or necrotizing fasciitis (Vezzulli et al., 2020). NTVC‐related infections are on the rise and represent one of the most striking examples of emerging human diseases linked to climate change (Newton et al., 2012; Vezzulli et al., 2020). In Europe, elevated numbers of NTVC‐related infections were primarily reported for the Baltic Sea during summer heat waves (Baker‐Austin et al., 2016; Brehm et al., 2021), but NTVC infections have been also increasingly reported for other European countries without connection to the Baltic Sea, such as the Netherlands (Engel et al., 2016; Schets et al., 2011), Belgium (De Keukeleire et al., 2018), France (Leroy et al., 2019), Croatia (Dobrovic et al., 2016) and Austria (Hirk et al., 2016) in connection with marine, marine‐influenced and in‐land recreational waters or connected to a private drinking water resource in Slovakia (Lackova & Sojka, 2018). In Austria, first documented cases were reported with the beginning of this century (Huhulescu et al., 2007) and their occurrence was documented in inland bathing waters in Eastern Austria (e.g. Lake Neusiedler See) (Kirschner et al., 2018; Schauer et al., 2015). Occurrence of NTVC in inland aquatic environments was also recorded in Eastern European countries such as Bulgaria (Eneva et al., 2011), Hungary (Szita et al., 1979), Poland (Stypulkowska‐Misiurewicz et al., 2006), Romania (Israil et al., 2003) or Slovakia (Valarikova et al., 2019), but knowledge on the environmental factors controlling NTVC occurrence and abundance in aquatic environments in non‐cholera epidemic regions are still scarce.

As high temperatures, elevated nutrients and a moderate salinity are known factors promoting *V. cholerae* growth (Schauer et al., 2015; Takemura et al., 2014) and based on the above reports from Eastern European countries, we hypothesized that NTVC may be commonly found in Serbian inland waters, an inland country in Eastern Europe, for which no official cases of NTVC infections were reported so far. Specifically, in the northern province of Vojvodina, where it is dry and hot during summer, natural and artificial lakes with elevated salinity, partly used for recreational purposes and fishing, exist. Thus, the aim of the study was a first surveillance of selected Serbian lakes and ponds in Vojvodina for the occurrence and abundance of *V. cholerae* with a culture‐based and a recently developed multiplex‐qPCR method. In addition, a representative set of 39 isolates was analysed for their genetic relatedness and pathogenicity factors by whole genome sequencing.

## EXPERIMENTAL PROCEDURES

**FIGURE 1:**
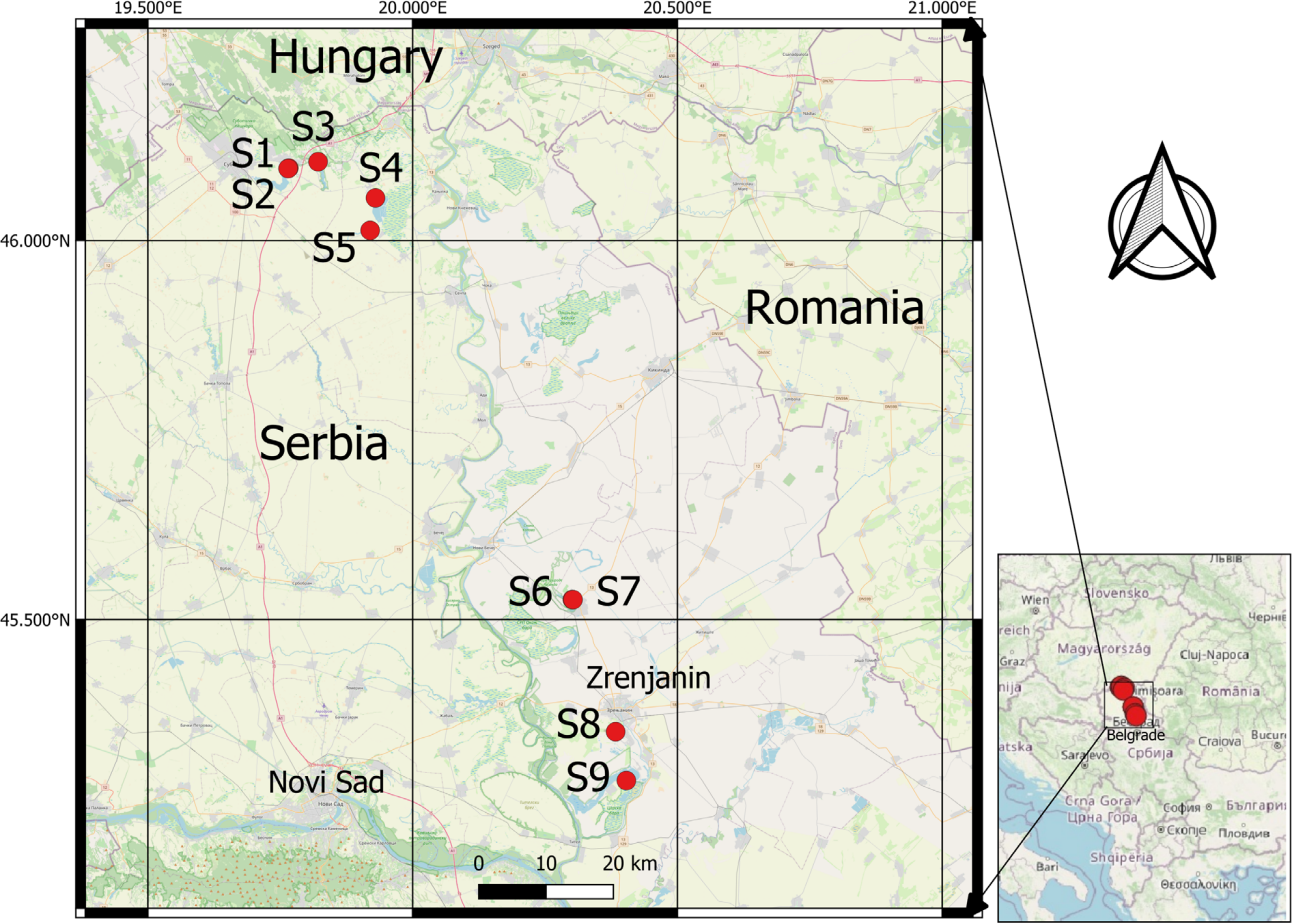
Geographical overview of the selected sampling sites S1–S9

In June 2021, nine natural and artificial lakes and ponds, most of them used for bathing and fishing, within the autonomous province of Vojvodina were sampled once during a period with water temperatures above 28°C (Figure 1). The lakes and ponds had been pre‐selected according to their salinity (electrical conductivity), displaying a gradient from hyposaline to saline environments. Water temperature, electrical conductivity, pH values and oxygen content were measured directly during sampling with a portable WTW multi‐parameter probe (Multi 3630 IDS Set F; WTW, Germany). Water samples were collected with a sampling rod in clean and sterile 500 ml glass bottles from a water depth of 30 cm, where possible. At site S6 (Rusanda jezero), the water level was too low (approx. 5 cm) and the water bottles had to be filled by hand. The samples were immediately transported to the laboratory in a dark cooling box at ambient temperature (approx. 25–30°C for a maximum of 4 h), to prevent a negative temperature effect on the culturability of *V. cholerae* and short enough to prevent additional growth (Alam et al., 2006).

*Vibrio cholerae* was determined by a membrane filtration approach recently developed for a variety of different bathing waters in Austria (Kirschner et al., 2018) and by a multiplex quantitative PCR (qPCR) approach, targeting total and toxigenic *V. cholerae*, recently applied in a large Eastern Austrian alkaline lake (Bliem et al., 2015; Bliem et al., 2018) with modifications. For cultivation‐based assessment, 0.1, 1, 10 and 100 ml volumes were filtered through 47 mm diameter nitro‐cellulose filters with a nominal pore size of 0.45 μm (Sartorius, Goettingen, Germany, Order no: 11406‐50), which were placed onto TCBS agar plates (Merck, Darmstadt, Germany, Ref. No: 1.10263.0500). After incubation at 37°C for 15 h, typical (yellow, 2–3 mm, round) colonies were counted to obtain the number of presumptive *V. cholerae*. For identification, 10 representative colonies were picked and transferred to Columbia nutrient agar without NaCl and incubated for another 24 h. All isolates growing on this agar were frozen for later identification via multiplex PCR (details see supplemental information SI). Based on these results, the weighted numbers of CFU per 100 ml were calculated from the different volumes.

To assess the genetic relatedness and potential pathogenicity, 39 representative isolates from all NTVC positive sites were selected and analysed via whole genome sequencing. Bacterial total DNA was isolated using the MagAttract HMW DNA Kit (Qiagen, Hilden, Germany), DNA concentration was determined with a Trinean DropSense16 (Unchained Labs, CA, USA) and libraries were prepared using the Nextera XT DNA Library Preparation Kit (Illumina, CA, USA) according to the manufacturer's instructions for whole genome sequencing. All isolates were paired‐end sequenced using NextSeq Reagent Kit P1 with a read length of 2 ✕ 150 base pairs on a NextSeq instrument (Illumina). SPAdes version 3.15.2 was used for de novo assembly of raw reads and further WGS data interpretation was carried out in the analysis software SeqSphere + (Ridom, Münster, Germany).

For multiplex‐qPCR, 30–100 ml of water sample depending on the turbidity of the sample was filtered through Isopore 0.2 μm pore size, 47 mm diameter polycarbonate filters (Merck Millipore, Darmstadt, Germany). The filters were aseptically folded several times, placed in 1.5 ml Eppendorf tubes and were immediately frozen at −20°C and (after transport of the filters to Austria) at −80°C until further processing. The DNA was extracted from the filters via a consecutive bead‐beating and phenol‐chloroform extraction (Griffiths et al., 2000; Linke et al., 2021). The total amount of bacterial DNA extracted was determined by a qPCR assay targeting the V1–V2 hypervariable region of the 16 S rRNA gene (denoted as 16 S‐rRNA qPCR; Savio et al., 2015, details see supplemental information S1). Toxigenic and non‐toxigenic *V. cholerae* were quantified by multiplex‐qPCR, targeting *ompW* for total and *ctxA* for toxigenic *V. cholerae* as well as *egfp*, as an internal amplification control to obtain quality‐controlled qPCR results (Bliem et al., 2015). All amplification reactions were performed in triplicates, using 2 μl of DNA template and including no‐template controls (NTC) in each run to check for contamination. To rule out qPCR inhibition, sample extract dilutions were additionally measured and judged free of inhibition based on matching concentrations. All controls, including no‐template controls as well as filtration and DNA extraction blanks (processed in parallel during sample filtration and DNA extraction), were consistently negative. Plasmid standards for all three target genes were used for the establishment of standard curves and the determination of the method limit of detection (five copies per reaction for all three targets). The sample limit of detection was determined, ranging between 125 and 416 (genomic units) GU per 100 ml. Details on the multiplex qPCR can be found in the Supplemental information SI. All qPCR assays in this study fulfilled the defined performance requirements: calculated PCR efficiency of 90%–105%, *R* ^2^ > 0.99 and standard deviations between replicates of <1 Ct. Results of qPCR are expressed in copies per ml (16 S‐rRNA qPCR) and as GU per 100 ml (*V. cholerae* multiplex‐qPCR).

For statistical analysis, IBM SPSS version 24 was used. Spearman‐rank correlations were calculated to identify potential causal relationships between the measured variables. A significance level of *p* < 0.05 was accepted as significant.

## RESULTS AND DISCUSSION

### Environmental conditions

During the sampling campaign, water temperatures of the investigated water bodies ranged between 28.0 and 35.9°C, representing favourable conditions for *V. cholerae* growth (Table 1). The salinity, measured as electrical conductivity, ranged between 500 μS/cm in Belo jezero (S9), a shallow fish and bathing lake, and 25.300 μS/cm in Rusanda jezero (S6), a shallow alkaline nature reserve lake that usually dries up completely during summer (Table 1). All water bodies displayed alkaline conditions with pH values ranging from 8.6 to 10.4. The highest pH was observed in a natural reserve lake (S3), coinciding with the highest oxygen concentrations of >20 mg/L, most likely due to intensive algal growth. Lowest oxygen concentrations occurred in Krvavo jezero (S1), a small nature reserve lake. In this lake, the highest number of 16S rRNA gene copies was detected, indicating a highly active bacterial community that obviously consumed a lot of oxygen. In general, 16 S rRNA gene copy numbers varied by three orders of magnitude from 1.2 × 10^5^ to 1.3 × 10^8^ copies per ml (Table 1).

**TABLE 1:** Sampling site descriptions, results of environmental variables and of *V. cholerae* concentrations determined via cultivation and multiplex‐qPCR

Code	Sampling site	Coordinates	Description	Temperature (°C)	Conductivity (μS/cm)	pH	O_2_ (mg/L)	16S‐rDNA (copies/ml)	*V. Cholerae* (CFU/100 ml)	*V. Cholerae‐ompW* (GU/100 ml)	*V. Cholerae‐ctx*A (GU/100 ml)
S1	Krvavo jezero/Lake Krvavo	46.0951, 19.7660	Natural aeolian lake, nature reserve	31.8	872	9.0	2.1	1.29 E+08	5.04 E+03	2.03 E+04	n.d.
S2	Palićko jezero/Lake Palić	46.0942, 19.7649	Natural aeolian lake, bathing lake	31.2	772	9.4	8.1	1.00 E+08	1.86 E+04	5.60 E+06	n.d.
S3	Ludosko jezero/Lake Ludos	46.1032, 19.8214	Natural aeolian lake, nature reserve	32.4	924	10.4	> 20	3.85 E+07	1.10 E+02	6.37 E+03	n.d.
S4	Jezero Kapitanski rit/Kireš River	46.0558, 19.9300	Connection of Ludoš and Kapetanski rit, fishing	31.4	1041	8.8	19.3	2.91 E+07	1.71 E+03	2.36 E+04	n.d.
S5	Rybnjak Velebit/Velebit pond	46.0130, 19.9198	Fish pond	29.6	991	8.6	10.7	1.19 E+05	8.10 E+02	4.51 E+02	n.d.
S6	Rusanda jezero/Lake Rusanda	45.5265, 20.3017	Alkaline soda lake, nature reserve	35.9	25,300	9.6	n.m.	1.00 E+06	n.d.	n.d.	n.d.
S7	small pond next to Lake Rusanda	45.5263, 20.3031	Bathing pond associated with Lake Rusanda	29.8	2590	8.9	11.7	8.23 E+06	7.00 E+02	1.26 E+04	n.d.
S8	Peskara jezero/Lake Peskara	45.3509, 20.3834	Artificial bathing pond	28.0	895	9.0	9.3	6.00 E+05	5.40 E+01	1.40 E+03	n.d.
S9	Belo jezero/Lake Belo	45.2864, 20.4038	Fluvial meander lake used for recreation and fishing	30.1	503	9.2	>20	2.46 E+07	5.30 E+03	6.25 E+04	n.d.

Abbreviations: n.d., not detected; n.m., not measured.

### Occurrence and abundance of NTVC

**FIGURE 2:**
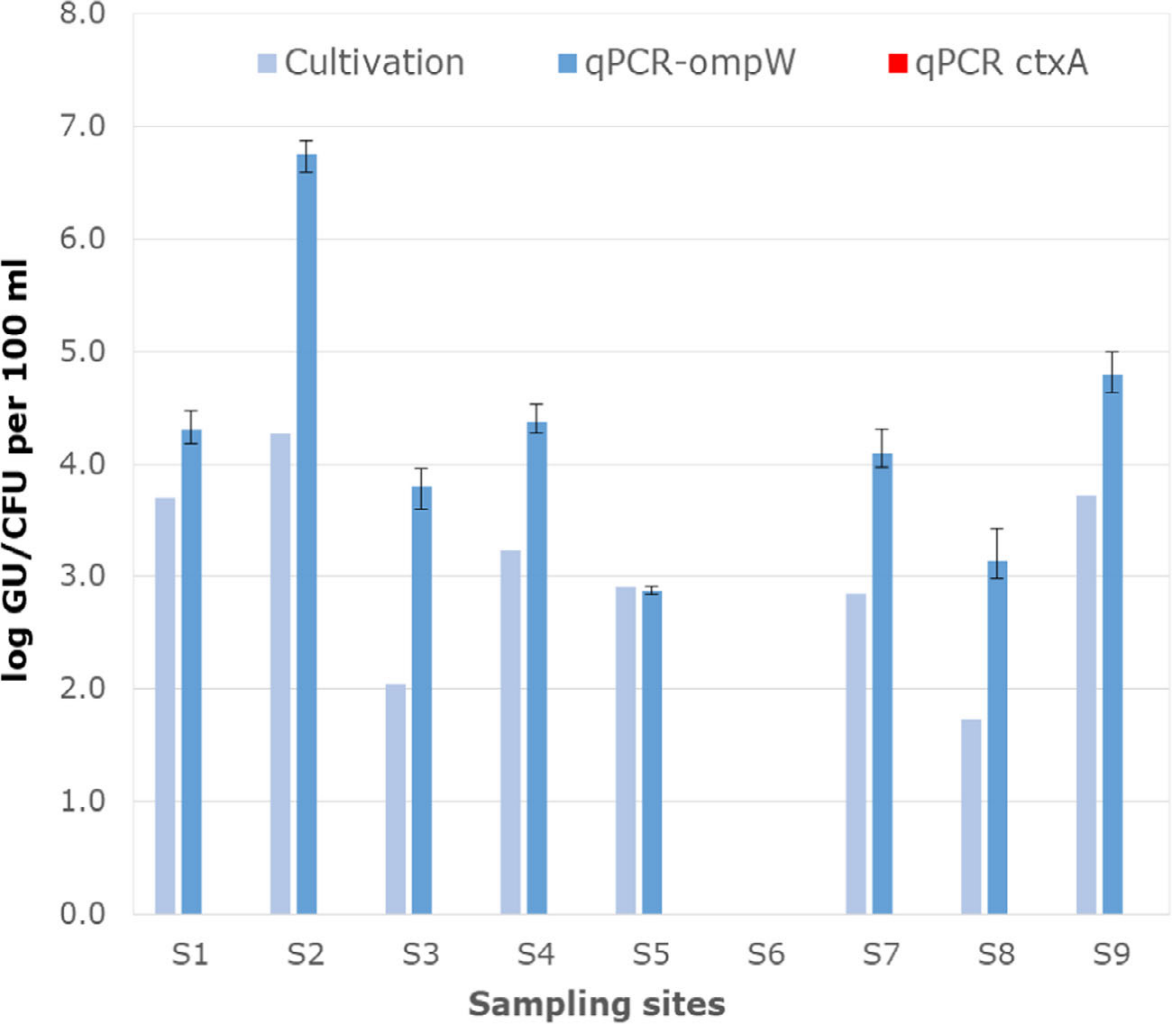
*Vibrio cholerae* concentrations determined via cultivation and multiplex qPCR. ompW: total *V. cholerae*, ctxA: toxigenic *V. cholerae* (no positive results). All data were expressed as log10 (*x* + 1), where *x* is the calculated concentration before applying the logarithm to it. qPCR results represent the geometric mean, error bars the minimum and maximum values of triplicate measurements.

With the exception of Rusanda jezero (S6), culturable NTVC were present in all investigated water bodies, ranging from 5.4 × 10^1^ to 1.86 × 10^4^ CFU per 100 ml (Table 1, Figure 2). All of the 71 colonies used for identification (those growing on Columbia nutrient agar without NaCl) had neither the *ctxA* or *tcpA* genes nor the markers for the serogroups O1 or O139. The only lake that did not harbour NTVC (Rusanda jezero, S6) was the lake with the highest salinity, which was sampled shortly before complete evaporation. The results obtained by the cultivation‐based method were confirmed by the multiplex qPCR. NTVC concentrations determined by qPCR generally ranged from 4.5 × 10^2^ to 5.6 × 10^6^ GU per 100 ml (Table 1, Figure 2). As for the cultivation‐based approach, no NTVC were detected in Rusanda jezero (S6). Likewise, no toxigenic *V. cholerae* was detected in any of the water bodies; no positive *ctxA* qPCR signal was recorded. Despite the fact that qPCR results were—with one exception—one to two orders of magnitude higher than culture‐based results, a highly significant correlation between the two methods was observed (Table 2; *rho* = 0.88, *p* < 0.01). The differences between cultivation‐based and qPCR‐based results are not surprising. This has been shown for NTVC in other ecosystems (Bliem et al., 2018) but also for other pathogens in different aquatic environments (Ditommaso et al., 2015; Räsänen et al., 2013). On the one hand, qPCR also detects dead and viable but non‐culturable (VBNC) cells as well as free DNA; on the other hand, colonies may emerge from more than one single cell, explaining the observed differences. The only sample (S5) with comparable *V. cholerae* results, 8.1 × 10^2^ (cultivation) vs 4.5 × 10^2^ (qPCR), was a fish pond that displayed the lowest 16 S rRNA gene copy numbers. Potentially, the low numbers were due to an inefficient DNA extraction, which can occur in specific environments with a high background turbidity, leading to an underestimation of qPCR results (Linke et al., 2021).

**TABLE 2:** Spearman rank correlation coefficients ρ and significance values (*p*‐value) of the correlations between the *V. cholerae* concentrations and environmental variables

Spearman‐rank correlation		Temperature (°C)	Conductivity (μS/cm)	pH	O2 (mg/L)	16 S‐rDNA (GU/ml)	*V. cholerae* (GU/100 ml)
*V. cholerae* (CFU/100 ml)	rho	−0.10	−**0.70**	−0.12	−0.20	0.58	**0.88**
	*p* value		**<0.05**				**<0.01**
*V. cholerae* (GU/100 ml)	rho	−0.02	−0.65	0.05	0.00	**0.70**	–
	*p* value					**<0.05**	

### Relationship of NTVC to environmental variables

For this set of selected water bodies in Serbia, culturable *V. cholerae* were significantly negatively correlated with conductivity (Table 2). The correlation of *V. cholerae* determined via qPCR with conductivity was slightly above the significance level of 0.05. It is known that *V. cholerae* preferably thrive in moderately saline environments (Takemura et al., 2014) and may be extinguished if salinity becomes too high, as observed for Rusanda jezero (S6). In a shallow alkaline lake in Austria, comparable to Rusanda jezero, conductivity was a significant factor controlling *V. cholerae* abundance (Schauer et al., 2015). During spring, the concentrations of *V. cholerae* increased with rising salinity and nutrient concentrations, due to continuing evaporation of the shallow lake water, but when salinity became too high, no more *V. cholerae* were detectable. Thus, the negative correlation observed here is due to the specific selection of the water bodies and not a general phenomenon. Due to the fact that temperature conditions were already in the optimal range for *V. cholerae* growth, no correlation of NTVC concentrations with temperature was observed. A similar argument applies to the pH values. Oxygen concentrations did not show a relationship to NTVC abundance. As *V. cholerae* is known to be facultative anaerobic (Youngren‐Grimes et al., 1988), oxygen is not a limiting factor and they were found in the Serbian lakes at oxygen concentrations ranging from nearly anaerobic to hyper‐saturated conditions. A significant correlation with 16S rRNA gene copy numbers was observed for *V. cholerae* determined via qPCR indicating that in more productive environments (with higher bacterial numbers) also larger NTVC concentrations exist.

### Genetic relatedness and pathogenicity factors of the isolates

All 39 sequenced isolates were positive for *ompW* (Table 3) and negative for *ctx*, *tcp* as well as for the serogroup O1 and O139 specific genes supporting the results of the multiplex PCR analysis. Interestingly, based on ribosomal multi‐locus sequence typing (rmlst), at sampling site S7 one isolate was identified as *V. paracholerae* (Table 3), a recently described closely related sister species of *V. cholerae* (Islam et al., 2021). In this small pond, also four other sequenced isolates were assigned with a high probability (84%–89%) as *V. paracholerae* and with only 4%–9% as *V. cholerae* (Table 3), indicating that in this specific pond, an abundant *V. paracholerae* population is present next to *V. cholerae*. One potential *V. paracholerae* isolate each was also found at sampling sites S4 and S8. So far, *V. paracholerae* has been isolated from Bangladesh surface waters but also from clinical and environmental specimens from North America, Thailand, Mozambique, Haiti and the United Kingdom, indicating that this sister species of *V. cholerae* is globally distributed (Islam et al., 2021). Concerning potential pathogenicity factors, all sequenced isolates were positive for *hlyA* (haemolysin; Manning et al., 1984). No isolate was positive for ace (accessory cholera enterotoxin), *zot* (zonula occludens toxin), *chxA* (cholix toxin) or *stn/sto* (heat‐stable enterotoxin) or *mshA*, the mannose‐sensitive haemagglutinin pilus, an important colonization factor (Jonson et al., 1991). Thirty‐two of the 39 isolates (82.1%) were positive for *toxR* (Provenzano et al., 2000), a major virulence regulator (Table 3). This widely present pathogenicity factor was not present in the *V. paracholerae* isolate and the isolate identified by rmlst as *V. paracholerae* with 91% probability. However, there were other isolates with a high probability (84%–89%) of being *V. paracholerae* that harboured *toxR* and *V. cholerae* isolates that did not harbour *toxR* (Table 3). This shows that both species cannot be separated from each other by these pathogenicity factors that are obviously shared and exchanged between the two species. Other pathogenicity factors found were *ompU* (23.1%), a major porine of the outer membrane supporting the colonization of the human intestine (Sperandio et al., 1995) and *makA* (Dongre et al., 2018), a motility associated killing factor and pore‐forming cytotoxin (17.9%), which was not present in the potential *V. paracholerae* isolates and *rtxA* (Lin et al., 1999), a toxin required for cytotoxic activity (15.4%; Table 3). In a recent study from Germany including isolates from the Baltic and the North Sea as well as clinical isolates, partly substantial differences in the frequency of pathogenicity factors in comparison to the Serbian isolates were observed (Schwartz et al., 2019). In that study *rtxA* and *ompU* were present in all whole genome sequenced isolates, while in our study, they were only present at rates between 15% and 23% (Table 3). Also *chxA* was present in five German isolates, while it was absent in all Serbian isolates.

**TABLE 3:** Overview of the pathogenicity factors identified via whole genome sequencing in the 39 analysed isolates and species assignment results from ribosomal multi‐locus sequence typing (rmlst)

ID	rMLST	ompW	hlyA	toxR	ompU	makA	rtxA
S1‐1	*V. cholerae*	+	+	+			
S1‐2	*V. cholerae*	+	+	+			
S1‐3	*V. cholerae*	+	+	+			
S1‐4	*V. cholerae*	+	+	+			
S1‐5	*V. cholerae*	+	+			+	
S1‐6	*V. cholerae*	+	+	+	+		+
S2‐1	*V. cholerae*	+	+	+			
S2‐4	*V. cholerae*	+	+	+			
S2‐5	*V. cholerae*	+	+	+			
S2‐6	*V. cholerae*	+	+	+			
S2‐8	*V. cholerae*	+	+	+			
S3‐1	*V. cholerae*	+	+	+			
S4‐1	*V. cholerae*	+	+	+			
S4‐2	87% *V. paracholerae*, 9% *V. cholerae*	+	+	+	+		
S4‐3	*V. cholerae*	+	+	+		+	
S4‐4	*V. cholerae*	+	+	+		+	
S4‐5	*V. cholerae*	+	+	+		+	
S5‐1	*V. cholerae*	+	+	+			
S5‐2	*V. cholerae*	+	+	+			
S5‐3	*V. cholerae*	+	+				
S5‐4	*V. cholerae*	+	+				
S5‐5	*V. cholerae*	+	+			+	
S7‐1	*V. cholerae*	+	+	+	+		+
S7‐2	87% *V. paracholerae*, 9% *V. cholerae*	+	+	+	+		
S7‐3	89% *V. paracholerae*, 4% *V. metoecus*	+	+	+			
S7‐4	*V. paracholerae*	+	+				
S7‐5	87% *V. paracholerae*, 9% *V. cholerae*	+	+	+	+		
S7‐6	84% *V. paracholerae*, 4% *V. cholerae*	+	+		+		
S8‐1	91% *V. paracholerae*, 4% *V. cholerae*	+	+				
S8‐2	*V. cholerae*	+	+	+			+
S8‐3	*V. cholerae*	+	+	+	+	+	+
S8‐4	*V. cholerae*	+	+	+			
S8‐5	*V. cholerae*	+	+	+	+		+
S8‐6	*V. cholerae*	+	+	+			
S9‐1	*V. cholerae*	+	+	+			
S9‐2	*V. cholerae*	+	+	+			
S9‐3	*V. cholerae*	+	+	+			
S9‐4	*V. cholerae*	+	+	+	+	+	+
S9‐5	*V. cholerae*	+	+	+			
# positive isolates		39/39	39/39	32/39	9/39	7/39	6/39
% positive isolates		100	100	82.1	23.1	17.9	15.4

*Note*: Only results with at least one positive isolate are shown. All isolates were negative for *ctx*, *tcp*, *rfbV*, *wbfZ*, *ace*, *zot*, *chxA*, *mshA* and *stn*/*sto*.

Abbreviations: ace, accessory cholera enterotoxin; chxA, cholix toxin; ctxA, toxigenic *V. cholerae*; mshA, mannose‐sensitive haemagglutinin pilus; stn/sto, heat‐stable enterotoxin; zot, zonula occludens toxi.

**FIGURE 3:**
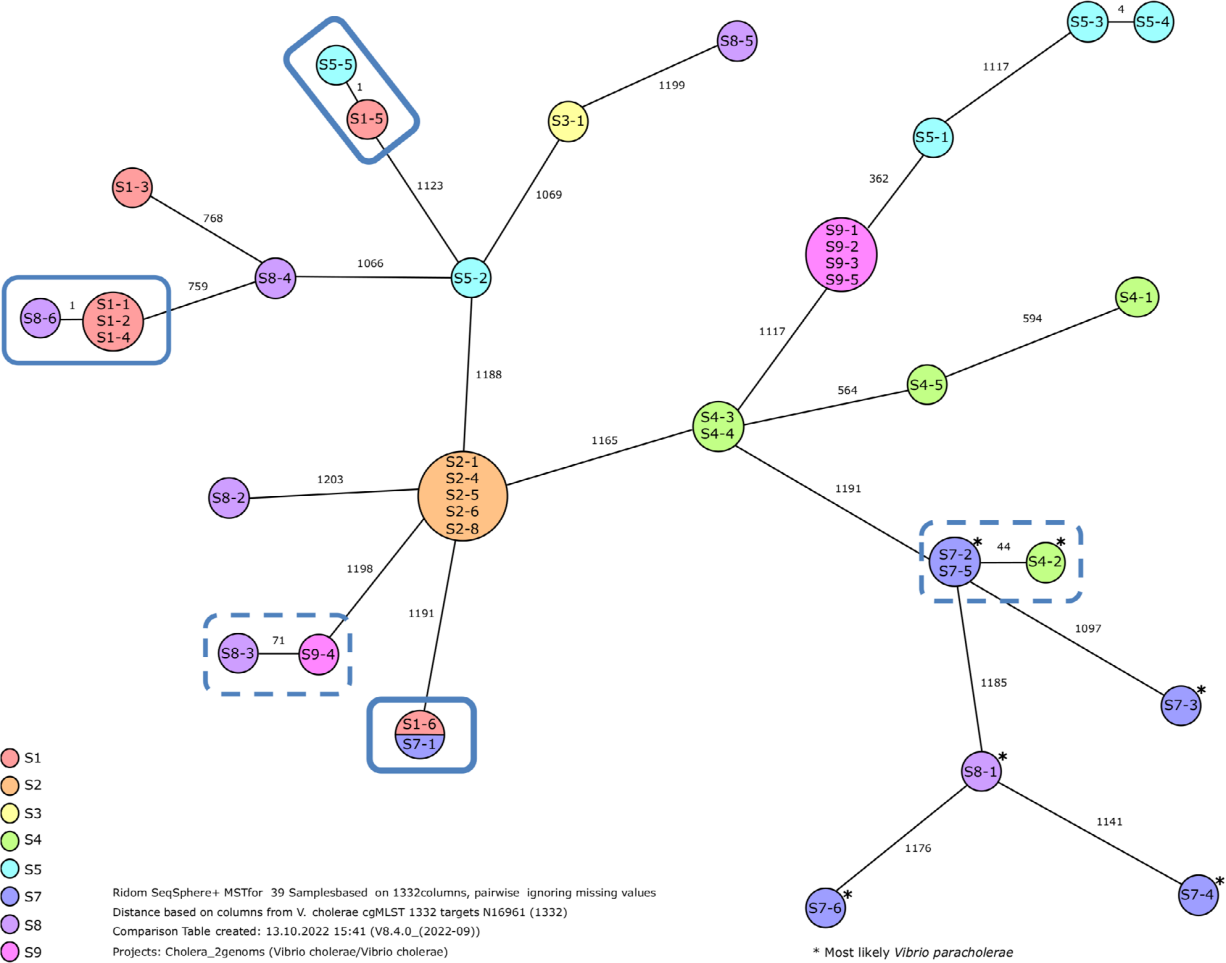
Minimum spanning tree based on cgMLST of the 39 analysed isolates. Circles represent the different genotypes containing the sequenced isolate numbers from the eight *V. cholerae* positive environments (S1–S9), represented by different colours. The numbers along the connecting lines represent the number of alleles that differ between the connected genotypes (from a total of 1332). Isolates with less than seven allelic differences are considered to belong to the same clone and isolates with less than 133 allelic differences belong to the same sub‐lineage (Liang et al., 2020). All potential *V. paracholerae* isolates determined via rMLST form a separate branch of the tree.

The minimum spanning tree showing the relatedness between the *V. cholerae* (and potential *V. paracholerae*) isolates based on core genome MLST (cgMLST) is depicted in Figure 3. With the exception of site S2, where all isolates belonged to a single clone, the isolates of the other lakes and ponds were largely spread over the whole tree. Specifically isolates from S8 were found in different branches of the tree. In addition, five sub‐lineages were found which consisted of identical (isolates S1‐6 and S7‐1) or near‐relative isolates from different ponds/lakes, differing in only 1 (isolates S1‐5 and S5‐5), 44 (isolates S7‐2, S7‐5 and S4‐2) or 71 (isolates S8‐3 and S9‐4) out of 1332 included genes. This clearly shows that isolates have been exchanged over long distances between the different investigated ecosystems in the region. As specifically the artificial bathing pond S8 that is intensively used for recreation had the most widely spread isolates, humans may be a significant factor for the regional transport of strains. Such intra‐continental transport of *V. cholerae* strains between aquatic ecosystems in Europe was already reported earlier by Pretzer et al (Pretzer et al., 2017), who hypothesized that mainly migrating birds or humans may be responsible for the observed phenomenon. One of the identified sub‐lineages was composed of isolates of the sites S4 and S7, being identified by rMLST with a high probability as *V. paracholerae*. Supporting the results of rMLST, all these potential *V. paracholerae* formed isolates clearly formed a separate branch of the tree (Figure 3).

### Comparison with inland waters in non‐cholera epidemic countries

Reliable numbers of *V. cholerae* in inland water bodies in non‐cholera epidemic countries are scarce. Vezzulli et al (Vezzulli et al., 2020) have compiled an overview of inland waters in their supplemental information, where concentrations based on cultivation and a culture‐independent, cell‐based assay were listed. Recent data (after year 2000) are from the Netherlands, Belgium, Austria and California, USA (De Keukeleire et al., 2018; Jiang, 2001; Kirschner et al., 2008, 2018; Schauer et al., 2015; Schets et al., 2011; Sterk et al., 2015), with concentrations up to 10^5^ CFU per 100 ml and up to 8 × 10^6^ cells per 100 ml, respectively. Another publication from Austria, where the same multiplex‐qPCR assay was applied, reported values of 6.2 × 10^5^ and 9.7 × 10^5^ GU per 100 ml for the saline lake Neusiedler See and two soda pools, respectively (Bliem et al., 2018). With maximum values of 2 × 10^4^ CFU per 100 ml for the culture‐based assay and 5.6 × 10^6^ GU per 100 ml for the multiplex‐qPCR assay, NTVC concentrations found in the Serbian lakes are in a similar order of magnitude, considering that only a single sample was taken and seasonal fluctuations were not considered in this study.

## CONCLUSIONS

With the established membrane filtration and multiplex‐qPCR assay, we were able to quantify culturable and total *V. cholerae* in a variety of ecologically different Serbian natural and artificial lakes and ponds. *Vibrio cholerae* were found in all selected water bodies, with the exception of the highly saline nature reserve Rusanda jezero, which was sampled shortly before complete evaporation. Obviously, *V. cholerae* are not able to cope with the highly saline and alkaline conditions present at the time of sampling. However, the high detection frequency of NTVC in the investigated water bodies in Serbia indicates the ubiquitous presence of NTVC in Eastern European moderately saline waters. Surprisingly, specifically in a small pond next to Rusanda jezero, the majority of isolates was identified as *V. paracholerae*, which was also found in two other lakes. With the exception of *makA*, pathogenicity factors such as *hlyA*, *toxR*, or *ompU* were present in both species indicating that both *V. cholerae* and *V. paracholerae* do have a relevant pathogenic potential. In the water bodies used as bathing ponds, culturable NTVC ranged from 54 to 18,600 CFU per 100 ml and total NTVC from 450 to 5.6 × 10^6^ indicating a quite large range of potential risks concerning the acquisition of an NTVC (or even a *V. paracholerae*) infection. As there are no established infection dose–response relationships for NTVC as regards wound infections, ear infections or gastrointestinal infections, the risks cannot be quantified. In Austria, mild‐to‐severe cases of NTVC infections (including lethal cases of septicaemia and necrotizing fasciitis; Hirk et al., 2016; Huhulescu et al., 2007) were so far only reported for recreational waters with rather high NTVC concentrations (10^4^–10^5^ CFU or MPN per 100 ml). One of these lethal cases was a fisherman under chemotherapy, who injured himself during fishing. If these numbers are adopted for basic risk estimation, sampling site S2 (Palićko jezero) would represent a bathing pond with a significant potential of an NTVC infection. However, even at lower NTVC numbers, susceptible persons (immunocompromised, chemotherapy patients) may be at risk during recreation and fishing if infection doses may be low. A seasonal sampling campaign in the investigated bathing and fishing ponds and other ponds/lakes used for recreation in Serbia with comparable physicochemical conditions are recommended to be prepared for a potential occurrence of infections promoted by climate change induced increase in bathing water temperatures. Physicians should be informed about potential risks from NTVC infections in connection with recreational activities in Serbian lakes and ponds. The fact that no such cases of NTVC infection have been reported in Serbia so far, does not mean that no infections have occurred, as the number of unreported cases may be substantial. NTVC infections are not a notifiable disease and physicians are mostly unaware of the possibility of an NTVC infection.

## AUTHOR CONTRIBUTION

AK, SK, MKK & AHF designed the study, CR, AK & CK wrote the manuscript, CR, SK, MKK, ML, SS, IS, LC and AR did the sampling and analysis of environmental, cultivation and qPCR parameters, KL and AI did the sequencing, sequence data interpretation and writing of sequencing results, all authors critically reveiwed the manuscript.

## CONFLICT OF INTEREST

The authors declare that they have no conflict of interest.

## Supporting information


**Table S1:** Primers for multiplex PCR
**Table S2:** Primers for 16 S‐rRNA qPCR
**Table S3:** Primers and probes for *Vibrio cholerae* multiplex qPCR (Bliem et al., 2015; Bliem et al., 2018)

## Data Availability

All data of this study are available in the publication and the supplemental information. Sequence data will be made available at public databases.
