# Supplementary material for: First report on the occurrence of Vibrio cholerae nonO1/nonO139 in natural and artificial lakes and ponds in Serbia: Evidence for a long‐distance transfer of strains and the presence of Vibrio paracholerae
Source: Environ Microbiol Rep. 2023 Feb 13;15(2):142–52. doi: 10.1111/1758-2229.13136 (PMC10103850; doi:10.1111/1758-2229.13136)
Supplement: Supplementary file 1 — Table S1: Primers for multiplex PCR Table S2: Primers for 16 S‐rRNA qPCR Table S3: Primers and probes for Vibrio cholerae multiplex qPCR (Bliem et al., 2015; Bliem et al., 2018) [file EMI4-15-142-s001.docx]

**Supplementary Information**

# First report on the occurrence of *Vibrio cholerae* nonO1/nonO139 in natural and artificial lakes and ponds in Serbia

Rehm C, Kolarević S, Kračun-Kolarević M, Leopold M, Steinbacher S, Schachner I, Campostrini L, Risslegger A, Farnleitner AH, Kolm C & Kirschner AKT

***Vibrio cholerae* multiplex PCR:**

*V. cholerae* multiplex PCR was used for analysis of presumptive *V. cholerae* isolates, as described previously ([Bliem et al., 2018](#_ENREF_1)). The isolates, grown on Columbia nutrient agar without NaCl were suspended in 1x PBS for DNA extraction. Cell lysis was performed by heating up the suspended colonies for 15 min at 95°C, followed by centrifugation at 13.000 × g. The supernatant was used as DNA template. Each PCR reaction was performed in a total reaction volume of 25 µL containing 5 µL of DNA template, 1x Solis Biodyne Taq Puffer (MgCl_2_ free Solis Biodyne, Estonia), 2 U µL^-1^ Solis Biodyne Hot DNA Polymerase (Solis Biodyne, Estonia), 2 µM MgCl_2_, 0.2 µM dNTPs, 480 nM of each of the forward and reverse primer of *ompW* and *tcp*, 360 nM of the *ctxA* primer pair and 400 nM of the rfbO1 and rfbO139 primer pairs. The corresponding primer sequences are given in **Supplementary Table S1**. Amplification reactions were performed on a T100 Thermal Cycler (BioRad, Germany) according to the following thermal cycling conditions: an initial denaturation at 94 °C for 15 min, followed by 30 cycles consisting of 94 °C for 1 min, 59 °C for 1 min and 72 °C for 2 min and a final extension step at 72 °C for 10 min. As a positive control, a mixture of a *V. cholerae* O139 and O1 strain was used. The PCR products were analyzed in 1.5% agarose gels (120 V, 400 mA, 100 min) stained with GelRed (Biotium, Fremont, CA) and visualized under UV light (Gel Doc, BioRad, USA).

**Table S1**: Primers for multiplex PCR

| Name/Target | Function | Sequence (5’-3’) | Amplicon size [bp] | Ref. |
| --- | --- | --- | --- | --- |
| *ompW* | Forward  Reverse | CAC CAA GAA GGT GAC TTT ATT GTG  GGT TTG TCG AAT TAG CTT CAC C | 304 | ([Goel et al., 2007](#_ENREF_6)) |
| *ctxAB* | Forward  Reverse | GCC GGG TTG TGG GAA TGC TCC AAG  GCC ATA CTA ATT GCG GCA ATC GCA TG | 536 | ([Goel et al., 2007](#_ENREF_6)) |
| *rfbO1* | Forward  Reverse | TCT ATG TGC TGC GAT TGG TG  CCC CGA AAA CCT AAT GTG AG | 638 | ([Goel et al., 2007](#_ENREF_6)) |
| *wbfO139* | Forward  Reverse | AGC CTC TTT ATT ACG GGT GG  GTC AAA CCC GAT CGT AAA GG | 449 | ([Das et al., 2009](#_ENREF_3)) |
| *tcpA* | Forward  Reverse | CGT TGG CGG TCA GTC TTG  CGG GCT TTC TTC TTG TTC G | 805 | ([Goel et al., 2007](#_ENREF_6)) |

**16S-rRNA qPCR:**

The 16S-rRNA qPCR was performed in a total reaction volume of 15 µL, containing 1x iQ SYBR Green Supermix (BioRad Laboratories, Hercules, USA), 2.5 µL of diluted DNA extract as template (1:10 and 1:100), 0.4 M BSA, 200 nM of primers 8F and 338R (targeting the V1-V2 region of bacterial 16S rRNA genes). Primer information is available in **Supplementary Table S2**. All qPCR reactions were performed on a qTOWER^3^ G cycler (Analytik Jena, Germany), according to the following thermal cycling conditions: initial denaturation at 95 °C for 3 min, followed by 40 cycles consisting of 95 °C for 30s, 57 °C for 30s and 72 °C for 1 min and a final extension step at 72 °C for 2 min. Additionally, a melting curve for each sample was recorded. Quantification was based on plasmid standard dilutions with known 16S rRNA gene copy numbers (10^8^-10^1^ copies), prepared in an unspecific background of 500 µg L^-1^ poly(dI-dC) (Roche Diagnostics, Mannheim, Germany). In each qPCR run, no-template controls were included to check for contamination. To rule out qPCR inhibition, sample extract dilutions (1:10 and 1:100) were measured and judged free of inhibition based on matching concentrations between the two dilutions.

**Table S2:** Primers for 16S-rRNA qPCR

| Name | Function | Sequence (5’-3’) | Amplicon size [bp] | Ref. |
| --- | --- | --- | --- | --- |
| *8F* | Forward | AGA GTT TGA TCC TGG CTC AG | 351 | ([Edwards et al., 1989](#_ENREF_4)) |
| 338R | Reverse | TGC TGC CTC CCG TAG GAG T |  | ([Etchebehere and Tiedje, 2005](#_ENREF_5)) |

***Vibrio cholerae* multiplex qPCR:**

The *V. cholerae* multiplex qPCR assay was performed in a total reaction volume of 20 µL, containing 1x Luna Universal Probe qPCR Master Mix (New England Biolabs, Ipswich, USA), 2 µL of DNA template (undiluted or 1:4-diluted extract), 200 nM of primers and probes, with exceptions for 150 nM ompW F1, 50 nM ompw F2, 100 nM egfp probe. Primer and probe sequences are listed in **Supplementary Table S3**. As an internal amplification control (IAC), the egfp plasmid was added at a concentration of 500 copies per reaction. Amplification reactions were performed on a qTOWER^3^ G cycler (Analytik Jena, Germany), according to the following thermal cycling conditions: initial denaturation at 94°C for 15 min, followed by 45 cycles consisting of 94°C for 15s, 60°C for 30s. Color compensation was performed according to the manufacturer’s instructions. Quantification was based on plasmid standard dilutions with known target gene copy numbers (10^8^-10^0^ copies), prepared in an unspecific background of 500 µg L^-1^ poly(dI-dC) (Roche Diagnostics, Mannheim, Germany) to avoid adsorption of plasmid DNA onto reaction tubes at low concentrations. In each qPCR run, no-template controls were included to check for contamination. To rule out possible qPCR inhibition, the results of both the IAC and the sample dilutions (undiluted and diluted 1:4) were assessed. Inhibition was assumed to be present, if the threshold cycle (Ct) of the IAC was shifted to higher Ct-values by more than one cycle in comparison to the mean of the Ct of the negative controls and/or the ratio of measured copy numbers (*ompW*/*ctxA*) deviated remarkedly from one (<0.5 or >2) after multiplication with the respective dilution factor.

**Table S3:** Primers and probes for *Vibrio cholerae* multiplex qPCR ([Bliem et al., 2015](#_ENREF_2); [Bliem et al., 2018](#_ENREF_1))

| Target | Function | Sequence (5’-3’) | Amplicon size [bp] | Purpose |
| --- | --- | --- | --- | --- |
| *ompW* | Forward1  Forward2  Reverse  Probe | AAG CTC CGC TCC TGT ATT TGC  ACT AGC CGC TCC TGT ATT TGC  GCT ATT AAC TGC CAA CTC ACT TTG AG  FAM-CAC CAA GAA GGT GAC TTT-MGB-NFQ | 127 | Total  *V. cholerae* |
| *ctxA* | Forward  Reverse  Probe | GCA TAG AGC TTG GAG GGA AGA G  CAT CGA TGA TCT TGG AGC ATT C  HEX-CAT CAT GCA CCG CCG-MGB-NFQ | 76 | Toxigenic  *V. cholerae* |
| *egfp* | Forward  Reverse  Probe | GAC CAC TAC CAG CAG AAC AC  GAA CTC CAG CAG GAC CAT G  TexasRed-AGC ACC CAG TCC GCC CTG AGC A-BHQ2 | 132 | IAC |

**References**

Bliem, R., Reischer, G., Linke, R., Farnleitner, A., and Kirschner, A.K.T. (2018) Spatiotemporal dynamics of Vibrio cholerae in turbid alkaline lakes as determined by quantitative PCR. *Applied and Environmental Microbiology* **84**.

Bliem, R., Schauer, S., Plicka, H., Obwaller, A., Sommer, R., Steinrigl, A. et al. (2015) A novel triplex quantitative pcr strategy for quantification of toxigenic and nontoxigenic *Vibrio cholerae* in aquatic environments. *Applied and Environmental Microbiology* **81**: 3077-3085.

Das, M., Bhowmick, T.S., Nandy, R.K., Nair, G.B., and Sarkar, B.L. (2009) Surveillance of vibriophages reveals their role as biomonitoring agents in Kolkata. *FEMS Microbiology Ecology* **67**: 502-510.

Edwards, U., Rogall, T., Blöcker, H., Emde, M., and Böttger, E.C. (1989) Isolation and direct complete nucleotide determination of entire genes. Characterization of a gene coding for 16S ribosomal RNA. *Nucleic Acids Research* **17**: 7843-7853.

Etchebehere, C., and Tiedje, J. (2005) Presence of two different active nirS nitrite reductase genes in a denitrifying Thauera sp. from a high-nitrate-removal-rate reactor. *Applied and Environmental Microbiology* **71**: 5642-5645.

Goel, A.K., Ponmariappan, S., Kamboj, D.V., and Singh, L. (2007) Single multiplex polymerase chain reaction for environmental surveillance of toxigenic - pathogenic O1 and non-O1 Vibrio cholerae. *Folia Microbiologica* **52**: 81-85.
